# Is Bigger Better? Living Donor Kidney Volume as Measured by the Donor CT Angiogram in Predicting Donor and Recipient eGFR after Living Donor Kidney Transplantation

**DOI:** 10.1155/2021/8885354

**Published:** 2021-07-09

**Authors:** Chaudhry Adeel Ebad, David Brennan, Julio Chevarria, Mohammad Bin Hussein, Donal Sexton, Douglas Mulholland, Ciaran Doyle, Patrick O'Kelly, Yvonne Williams, Ruth Dunne, Conall O'Seaghdha, Dilly Little, Martina Morrin, Peter J. Conlon

**Affiliations:** ^1^Department of Nephrology and Kidney Transplantation, Beaumont Hospital, Dublin, Ireland; ^2^Department of Radiology, Beaumont Hospital, Dublin, Ireland; ^3^Department of Urology and Kidney Transplantation, Beaumont Hospital, Dublin, Ireland

## Abstract

**Background:**

The role of kidney volume measurement in predicting the donor and recipient kidney function is not clear.

**Methods:**

We measured kidney volume bilaterally in living kidney donors using CT angiography and assessed the association with the donor remaining kidney and recipient kidney (donated kidney) function at 1 year after kidney transplantation. Donor volume was categorized into tertiles based on lowest, middle, and highest volume.

**Results:**

There were 166 living donor and recipient pairs. The mean donor age was 44.8 years (SD ± 10.8), and donor mean BMI was 25.5 (SD ± 2.9). The recipients of living donor kidneys were 64% male and had a mean age of 43.5 years (SD ± 13.3). Six percent of patients experienced an episode of cellular rejection and were maintained on dialysis for a mean of 18 months (13–32) prior to transplant. Kidney volume was divided into tertiles based on lowest, middle, and highest volume. Kidney volume median (range) in tertiles 1, 2, and 3 was 124 (89–135 ml), 155 (136–164 ml), and 184 (165–240 ml) with donor eGFR ml/min (adjusted for body surface area expressed as ml/min/1.73 m^2^) at the time of donation in each tertile, 109 (93–129), 110 (92–132), and 101 ml/min (84–117). The median (IQR) eGFR in tertiles 1 to 3 in kidney recipients at 1 year after donation was 54 (44–67), 62 (50–75), and 63 ml/min (58–79), respectively. The median (IQR) eGFR in tertiles 1 to 3 in the remaining kidney of donors at 1 year after donation was 59 (53–66), 65 (57–72), and 65 ml/min (56–73), respectively.

**Conclusion:**

Bigger kidney volume was associated with better eGFR at 1 year after transplant in the recipient and marginally in the donor remaining kidney.

## 1. Background

End-stage kidney disease is associated with significant mortality and morbidity [1]. Living donor kidney transplantation, where available, is the treatment of choice in end-stage kidney disease [2].

In 1992, Brenner and coworkers described the concept of nephron dosing. The delivery of an adequate number of nephrons in accordance with recipient metabolic needs has a significant impact on post-transplant recipient outcomes[3]. According to this theory, smaller kidneys will have a lower number of nephrons and a consequent negative impact on the graft outcome and function in recipients [4].

The shortage of deceased organ donors and resultant increased deceased donor wait-list time have perhaps resulted in an increased focus on living kidney donors. Living kidney donor recipients tend to enjoy not only shorter wait times but also superior graft outcomes in comparison to recipients of deceased donor kidneys [5, 6].

The assessment of living donors involves rigorous investigations [7, 8] including an assessment of estimated glomerular filtration rate (eGFR) from creatinine-based equations, creatinine clearance from urine collection [9], and, more recently, nuclear medicine-based measured glomerular filtration rate (mGFR) estimation [10]. In addition, computerized tomographic renal angiography (CTA) is performed routinely in donor evaluation, with magnetic resonance angiography (MRA) representing an optional additional assessment [11–14].

A small number of previous studies have assessed the association between kidney volume measured by CTA, magnetic resonance angiography (MRA), and ultrasound (US), and variables including donor body weight, donor BMI, recipient body weight, recipient BMI, and body surface area are assessed to predict the recipient graft outcome [15, 16]. In addition, a limited number of studies have reported an association between remaining kidney volume after donation and donor eGFR [17, 18].

In this analysis, we set out to determine the utility of CT-derived donor kidney volume in predicting eGFR in the donor remaining kidney and recipient kidney at 1 year after kidney transplantation.

## 2. Materials and Methods

This was a retrospective cohort study of living kidney donors and recipients (2012–2017) from the National Kidney Transplant Service (NKTS) center at Beaumont Hospital. Living kidney donors and recipients above 18 years of age were selected for inclusion. As a part of the NKTS standard living donor evaluation protocol, all potential kidney donors undergo a CT angiogram to assess the renal structure and arterial and venous anatomy in addition to extensive medical and biochemical evaluation [11].

The Chronic Kidney Disease Epidemiology Collaboration (CKD-EPI) equation was used to calculate eGFR in the kidney donor prior to donation and at 1 year after transplantation for both donor and recipient. Approval for the study was obtained from the local research ethics committee.

The clinical and research activities being reported are consistent with the principles of the Declaration of Istanbul as outlined in the ‘Declaration of Istanbul on Organ Trafficking and Transplant Tourism' and the Declaration of Helsinki.

### 2.1. Kidney Volume Measurement

**Figure 1:**
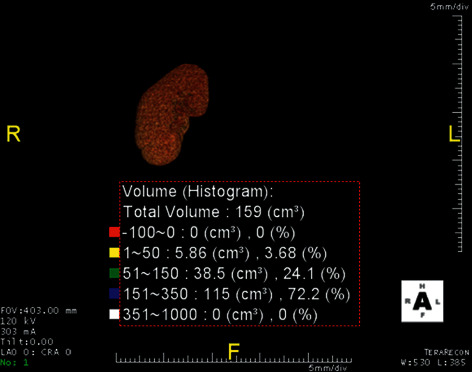
Reconstructed image shows right kidney volume histogram attenuation with software.

**Figure 2:**
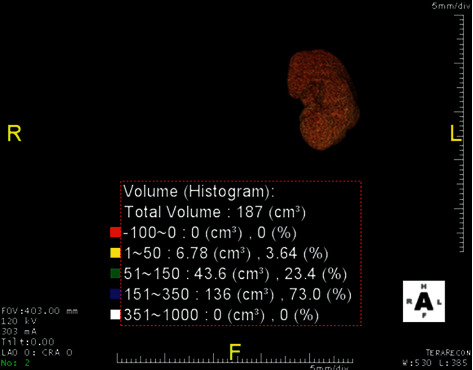
Reconstructed image shows left kidney volume histogram attenuation with software.

Donor kidney CT angiogram protocol at this institution is a low-dose noncontrast CT (typically 45–50 mAs) from xiphisternum to the pubic symphysis with a hand injection of 50 ml of iopamidol (Niopam 300, Bracco UK Limited), 10-minute delay, and 100 ml of the iopamidol (Niopam 370, Bracco UK Limited) pump injected at 5 ml/s bolus tracked; images from the xiphisternum to the pubic symphysis result in combined arterial and urographic phase CTA, reconstructed with a body soft-tissue algorithm in 1 mm- and 3 mm-thick sections with axial, sagittal, and coronal reformations. Kidney volume on both kidneys of the living donor from processed images was measured with TeraRecon USA standard customized software using 1 mm arterial phase sequence from the CT angiogram, 3D image of the kidney with threshold attenuation applied of 10–350 Hounsfield Units double was read by two experienced radiologists. The reconstructed images of both kidneys of the donors are shown in Figures 1 and 2.

### 2.2. Statistical Analysis

Donor and recipient demographic results for the tertile groups were assessed using either ANOVA or Kruskal–Wallis tests for continuous data or Fisher's exact tests for categorical data. Correlation coefficients between donor kidney volume and 1-year donor remaining kidney and recipient eGFR were calculated and presented graphically.

Linear regression models were constructed to predict log of 1-year eGFR with kidney volume adjusted for potential confounding variables including age, sex, HLA mismatch >3, acute rejection episodes, and BMI. All analyses were conducted using Stata/SE (version 13, College Station, Texas). Results were deemed to be significant where the probability of a type 1 error (*P* value) was less than 0.05.

## 3. Results

Over a 5-year period, 166 living donors for whom there were complete clinical data and kidney volume imaging available and who went on to successfully donate a kidney were evaluated. Each donor and recipient pair had at least 1-year follow-up data available. The demographic characteristics of donors and recipients are shown in Table 1.

The kidney volume was divided into tertiles based on lowest, middle, and highest volume. The kidney volume median (range) in tertiles 1, 2, and 3 was 124 (89–135), 155 (136–164), and 184 ml (165–240) with donor eGFR at the time of donation in each tertile, 109 (93–129), 110 (92–132), and 101 ml/min (84–117), respectively.

Tertile group 1 included the highest percentage of oldest donor and recipient ages. In addition, this tertile had the lowest percentage of male donors (16%).

**Figure 3:**
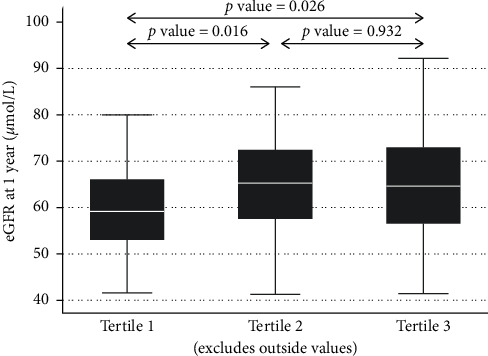
Donor eGFR and donor kidney volume by tertile at 1 year after donation.

The median (IQR) eGFR in tertiles 1 to 3 in the remaining kidney of donors at 1 year after donation was 59, 65, and 65 ml/min (56–73), respectively (Figure 3).

**Figure 4:**
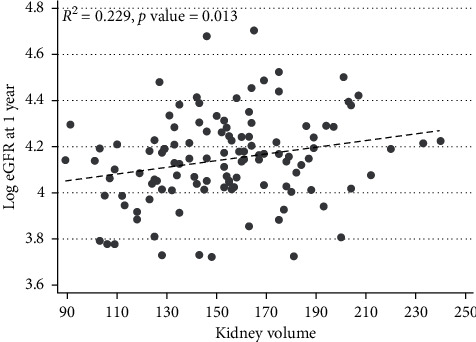
Correlation of kidney volume and eGFR in kidney donors at 1 year after donation.

The association between predonation kidney volume and donor remaining kidney eGFR at 1 year is displayed in Figure 4 and shows a weak and marginal correlation effect.

Multivariable modelling of the association of donor characteristics and donor remaining kidney eGFR at 1 year is displayed in Table 2. After adjustment for multiple donor characteristics, donor remaining kidney volume baseline reference tertile 1 relative to tertile 2 was not significantly associated with donor eGFR at 1 year after donation but relative to tertile 3 was marginally significant.

**Figure 5:**
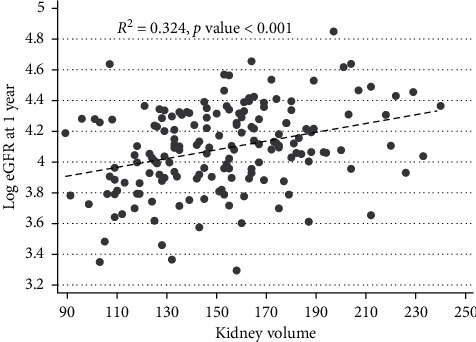
Correlation of kidney volume and eGFR in kidney transplant recipients at 1 year after transplant.

The correlation between donated kidney volume and recipient eGFR at 1 year after transplantation is shown in Figure 5 and shows a moderate *R*^2^ statistic with a highly significant *P* value.

**Figure 6:**
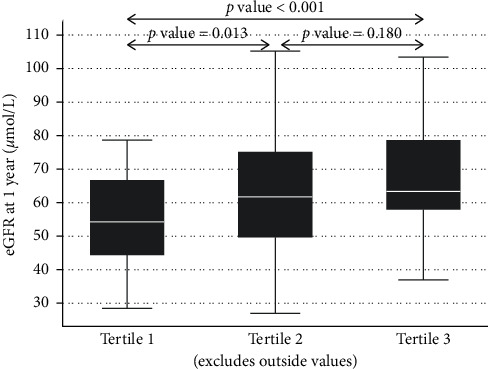
Recipient eGFR and donor kidney volume by tertile at 1 year after transplant.

The median (IQR) eGFR in tertiles 1 to 3 in kidney recipients at 1 year after donation was 54 (44–67), 62 (50–75), and 63 ml/min (58–79), respectively (Figure 6).

Multivariable modelling of the association of donor and recipient characteristics with recipient eGFR at 1 year is displayed in Table 3. After adjustment for donor and recipient characteristics, donor kidney volume baseline reference tertile 1 relative to tertile 2 was not significantly associated with recipient eGFR at 1 year after donation but relative to tertile 3 was highly significant with recipient eGFR.

Predonation kidney volume as measured by CT angiography showed a moderate association with eGFR in recipients at 1 year after transplant but had a weaker effect on donor eGFR.

## 4. Discussion

The nephron mass of a transplanted kidney has an important impact on post-transplant outcomes [19]. In contrast to deceased donor transplantation, in living donor kidney weight which cannot be measured before donation, kidney volume can be estimated before donation and can act as a surrogate marker of nephron mass in a living donor kidney [20]. In this study, bilateral total kidney volume for living donors was measured from CT angiography by expert radiologists.

Sometimes, transplant surgeons have a choice of different living donors to utilise for a given potential transplant recipient. Common clinical factors that are utilised to assess the suitability of a given donor include donor age, HLA mismatch, donor weight, and kidney vascular anatomy. In this analysis, we have demonstrated that donor kidney volume should also be considered when these other factors are equivalent. Where a larger kidney is available for transplantation, it is likely that the recipient will be left with a higher GFR at 1 year.

There are significant data demonstrating an association between GFR at 1 year and long-term graft function, so it is appropriate that efforts are made to maximise GFR at 1 year after transplantation.

In contrast to the deceased donor kidney transplant scenario, previous studies have suggested that living kidney volume could represent an independent determinant of posttransplant kidney function [21]. Factors such as donor comorbidities, cause of death, and cold ischemia time may confound the association between deceased donor kidney size on allograft function [22, 23].

Hawley and Nicol, in a small retrospective study of 45 living donors, were not able to show that CT-measured kidney volume predicts short-term graft outcome at 6 months after living donor kidney transplantation [24].

Other investigators have previously studied the association of renal volume and posttransplant GFR. Poggio et al. studied 104 donor and recipient pairs which showed a significant correlation of kidney volume to determine posttransplant GFR [21]. Dias et al. and Juluru et al. made similar observations with Juluru et al. extending this observation on GFR to 24 months after transplantation [15, 25].

When considering living donors, transplant surgeons need to not only consider the likely GFR the recipient will be left with but also the impact of nephrectomy on the donor, as principle surgeons generally prefer to leave the donor with the better kidney when there are discrepancies in kidney quality between each kidney. In our study, findings showed that not only is the donated living kidney volume independently associated with recipient eGFR at 1 year after transplant but also the donor remaining kidney is marginally associated with the donor eGFR at 1 year after transplant.

Limitations of this study include its retrospective nature, the lack of actual measured GFR, and unadjusted eGFR with BMI. In addition, although the CKD-EPI equation is comparably better to other equations in the transplant population [26, 27], it has not been specifically validated in the kidney transplantation population. Cystatin C-based calculations are emerging as better predictors of eGFR measurement and may merit future study in this context [28, 29]. Although our study has some limitations, we believe our findings are relevant to the choice of kidney for transplantation and support the emerging concept of bigger kidney volume as a predictor of better recipient outcomes.

## Figures and Tables

**Table 1:** Demographic characteristics of donors and recipients and variables.

Recipient and donor variables	Overall	Tertile 1	Tertile 2	Tertile 3	*P* value
Donor age, mean (SD)	44.8 (10.8)	49.3 (11.0)	42.1 (11.6)	42.7 (8.2)	<0.001
Donor sex (% male)	43	16	32	85	<0.001
Donor BMI, mean (SD)	25.5 (2.9)	24.9 (2.8)	25.0 (2.9)	26.7 (2.5)	0.001
Donor eGFR at Tx^*∗*^	107 [90–125]	109 [93–130]	114 [94–141]	101 [85–116]	0.023
Donor eGFR at 1 yr	63 [56–70]	59 [53–66]	65 [57–72]	65 [56–73]	0.028
Recipient eGFR at 1 yr	60 [50–73]	54 [44–67]	62 [50–75]	63 [58–79]	<0.001
Recipient age	43.5 (13.3)	45.3 (14.7)	43.2 (13.2)	41.8 (11.6)	0.367
Recipient sex (% male)	64	69	61	61	0.604
Acute rejection	6.6	3.5	13.0	3.7	0.074
HLA mismatch > 3	21.7	27.6	18.5	18.5	0.401
PRA groups (0–10/11–49/50–84/85+)	28/36/20/16	38/28/22/13	19/44/22/15	26/35/17/22	0.199
Recipient BMI	25.2 (4.0)	24.6 (3.6)	24.5 (4.1)	26.5 (4.1)	0.020
Recipient weight	74.9 (15.6)	74.0 (13.6)	73.9 (15.2)	76.7 (17.8)	0.558
Time on dialysis vintage (months)	18 [13–32]	22 [14–31]	18 [11–30]	18 [15–35]	0.518

ABO compatibility					
ABO-A	29 (17.6)	8 (13.8)	8 (15.1)	13 (24.1)	0.301
ABO-B	8 (4.9)	1 (1.7)	3 (5.7)	4 (7.4)	
ABO-O	128 (77.6)	49 (84.5)	42 (79.2)	37 (68.5)	

^*∗*^Tx means transplant. Donor eGFR at Tx means eGFR from both kidneys of the donor before donation. Donor eGFR at 1 year means eGFR from the donors' remaining kidney. Recipient eGFR at 1 year means eGFR of the donated kidney.

**Table 2:** Multivariable model for the effect of donor volume tertiles on 1-year donor GFR.

Variable	Coefficient	95% conf. interval	*P* value
Tertile			
1	0.000	—	—
2	0.040	−0.032–0.112	0.271
3	0.102	0.013–0.192	0.026

Donor BMI	−0.021	−0.032–0.011	<0.001
Donor age	−0.009	−0.012–0.006	<0.001
Donor sex	−0.016	−0.089–0.057	0.664
Constant	5.068	4.779–5.356	<0.001

**Table 3:** Multivariable model for the effect of donor volume tertiles on 1-year recipient GFR.

Variable	Coefficient	95% conf. interval	*P* value
Tertile			
1	0.000	—	
2	0.079	−0.023–0.175	0.133
3	0.221	0.106–0.337	<0.001

Recipient BMI	−0.013	−0.023–0.004	0.007
Donor age	−0.012	−0.016–0.008	<0.001
Donor sex	0.073	−0.169–0.023	0.135
Acute rejection	−0.150	−0.305–0.005	0.057
PRA group	−0.005	−0.043–0.032	0.763
HLA >3 mm	0.106	0.007–0.206	0.037
Constant	4.970	4.606–5.335	<0.001

## Data Availability

Data are not available.

## Abbreviations

SD: Standard deviation
IQR: Interquartile range
eGFR: Estimated glomerular filtration rate
GFR: Glomerular filtration rate
mGFR: Measured glomerular filtration rate
CT: Computerized tomography
CTA: Computerized tomographic angiography
3D: Three-dimensional
SE: Special edition
BMI: Body mass index
BSA: Body surface area
MRA: Magnetic resonance angiography
CKD-EPI: Chronic Kidney Disease Epidemiology Collaboration
Tx: Transplantation
ESRD: End-stage renal disease
ABO: Blood groups A, B, and O
PRA: Panel reactive antigen
HLA mismatch: Human leukocyte antigen mismatch
US: Ultrasound
ANOVA: Analysis of variance
USA: United States of America
UK: United Kingdom
NKTS: National Kidney Transplant Service
ml/min/ 1.73 m^2^: Millilitres per minute relative to body surface area
ml: Millilitres
ml/s: Millilitres per second
mAs: Milliampere-seconds.
